# Experimental Investigation of the Applicability of the Stress‐Based and Strain‐Based Hemolysis Models for Short‐Term Stress Peaks Typical for Rotary Blood Pumps

**DOI:** 10.1111/aor.15002

**Published:** 2025-04-16

**Authors:** Michael Lommel, Vera Charlotte Lommel, Henri Wolff, Nico Dirkes, Katharina Vellguth, Marek Behr, Ulrich Kertzscher

**Affiliations:** ^1^ Biofluid Mechanics Laboratory Institute of Computer‐Assisted Cardiovascular Medicine, Deutsches Herzzentrum der Charité Berlin Germany; ^2^ Charité—Universitätsmedizin Berlin, corporate member of Freie Universität Berlin, Humboldt Universität zu Berlin Berlin Germany; ^3^ Chair for Computational Analysis of Technical Systems RWTH Aachen University Aachen Germany

**Keywords:** CFD, experiments, flow channel, hemolysis, hemolysis model, rotary blood pumps

## Abstract

**Background:**

Although flow simulations have become more accurate, hemolysis prediction models still show large deviations from measured values in blood‐carrying devices. To develop and validate more accurate models, specific hemolysis experiments are needed to determine the influence of parameters on hemolysis, such as stress type and exposure time typical for rotary blood pumps (RBPs).

**Methods:**

In order to investigate the applicability of the hemolysis models to the flow in RBPs, this study performed experiments with human whole blood in three differently shaped flow channels that generate short‐term stress peaks. CFD simulations were performed and the applicability of the stress‐based and strain‐based hemolysis models was investigated by comparing them with the experimental results.

**Results:**

Low and statistically non‐significant hemolysis was measured for all geometries and 1200 load repetitions. The CFD simulations determined scalar shear stresses up to 1500 Pa with exposure times in the millisecond range. The stress‐based hemolysis model overestimated hemolysis by several orders of magnitude and predicted significant differences between the three flow channels. The strain‐based hemolysis model predicted low and approximately equal hemolysis, in agreement with the experimental results.

**Conclusion:**

The results suggest that the stress‐based hemolysis model is not appropriate for the applied short‐term stress peaks. The strain‐based model, which considers the deformation of the RBCs over time, appears to be more appropriate for this type of flow. This implies a similar relationship for RBPs, where these short‐term stress peaks are typical.

## Graphical abstract

Human blood hemolysis experiments with microchannels (I, II, III) were compared with simulations (A, B) by applying stress‐based and strain‐based hemolysis models. The stress‐based model showed a low applicability for short‐term stress peaks, while the strain‐based model, which considers the time‐dependent deformation of RBCs, shows more promising results.

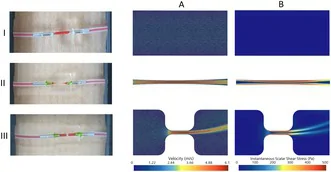

## 1 Introduction

The measurement and reduction of flow‐induced damage to red blood cells (RBCs), known as hemolysis, plays a central role in the development and comparison of blood‐carrying medical devices such as rotary blood pumps (RBPs). To determine the hemolysis caused by a device, the increasing concentration of plasma free hemoglobin (pfHb) over time is measured in vitro in a test circuit. In the development process of new devices, it is important to predict hemolysis at an early stage. To achieve this, several models have been developed to predict hemolysis based on the flow field obtained from computational fluid dynamics (CFD) simulations [1].

With the commonly used stress‐based models, the prediction of hemolysis in RBPs is still inaccurate and the validity range remains unclear, as there is no computational model that has been sufficiently validated for more complex flow conditions [1, 2]. The lack of confidence in the models and the difficulty of implementation is also reflected in the FDA benchmark investigation for medical device flow models for CFD validation, where only 19 of the 52 participating groups implemented a hemolysis model [3]. In another interlaboratory CFD study of the FDA benchmark RBP, only one of the eight participants who submitted hemolysis results accurately predicted absolute pfHb levels under the majority of conditions, despite relatively inaccurate predictions of the flow field [4].

The input variables in the stress‐based hemolysis models are a scalar measure of fluid stress *τ* and the exposure time *t*, from which a damage index *DI* is calculated using a power function [2]:

(1) $$\mathrm{DI}\left(\tau ,t\right)=C\times t^{\alpha }\times \tau^{\beta },$$

where *C*, *α* and *β* are parameters, which have been determined experimentally and showed non‐linear dependencies with *α* < 1.0 and *β* > 1.0 [1, 5].

The parameters for these models are based primarily on experiments using Couette shearing devices, which exposed blood to shear flow in the range of 30 to 850 Pa within exposure times of 0.025 to 120 s [2, 6].

Alternatively, there are models that calculate the membrane tension and the resulting deformation and elongation of the RBCs based on the local stress tensor to estimate hemolysis. These models are usually referred to as strain‐based models and have been investigated and applied in fewer studies [1, 2, 7, 8, 9]. They take into account the mechanical properties of RBCs, such as the relaxation time, which was derived from deformation experiments. The correlation for hemoglobin release is then applied to the calculated membrane strain.

In addition to Couette shearing devices, hemolysis experiments have also been performed in flow channels to study deformation and damage to RBCs [10]. Factors influencing damage that are not considered in stress‐based models have been identified, such as an increase in damage threshold with shorter exposure times and the differential influence of different types of stress [2, 11, 12]. In their review, Faghih and Sharp [2] summarize the current state and challenges of hemolysis models and highlight the high demand for in vitro hemolysis measurements in extensional laminar flow at a hemolytic level for hemolysis modeling, as previous studies focused on shear stress rather than extensional (normal) stress. Mantegazza et al. [13] have shown that the stress‐based hemolysis model is generally not suitable for evaluating the absolute hemolytic potential of a medical device, even with new parameters calibrated to similar flow cases.

To further investigate this and to address this knowledge gap, we have developed a test setup using high precision, optically accessible flow channels that allow the study of hemolysis in a flow field with short‐term stress loads. To include the effect of previous sub‐lethal damage, the blood is exposed to superphysiological stresses up to 1200 times over a period of 6 h during each experiment. The experimental setup was designed and optimized to minimize blood damage outside the channels. This was achieved by using an in‐line setup with no flow deflections or sharp transitions between the components.

Three different flow channels were designed to distinguish the influence of extensional (elongational) stresses from shear stresses: one with a sudden constriction with high extensional and high shear stresses, one with a smooth constriction with low extensional and high shear stresses, and one reference channel without any constriction with low extensional and low shear stresses. The geometry and flow conditions are based on the blade gap region of the most frequently clinically implanted RBP, the HeartMate II (Abbott Laboratories, Chicago, USA), since, based on the hemolysis models, this region is thought to be responsible for most of the hemolysis that occurs [14].

CFD simulations of all three geometries were performed to apply the hemolysis models and compare the results to the measured hemolysis. The aim was to test the applicability of the different hemolysis models to short‐term stress peaks typical for RBPs [15].

## 2 Materials and Methods

### 2.1 Fluidic Set Up

**FIGURE 1:**
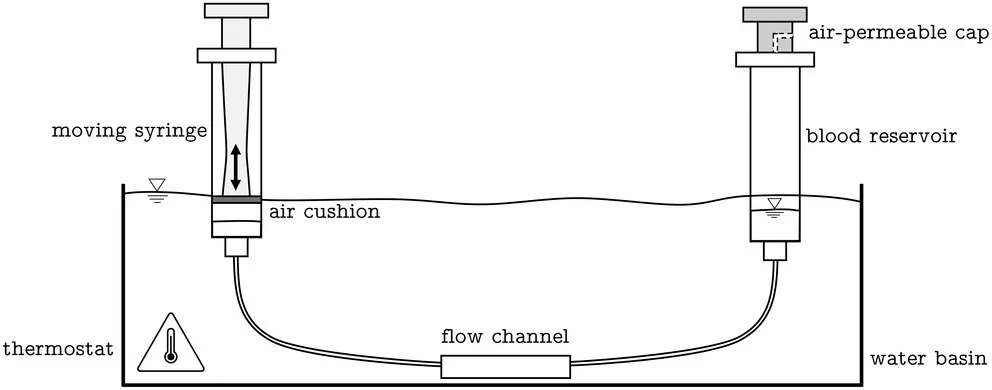
Schematic of the experimental setup with one flow channel: The blood is pumped by a syringe pump back and forth through the channel. An air cushion is implemented to reduce hemolysis in the syringe. Filling of the test section and sampling during the experiment are performed via the blood reservoir, which is equipped with a removable and air‐permeable cap.

The schematic setup is shown in Figure 1. All blood‐bearing components are located in a water basin that is temperature‐controlled by a thermostat to 36°C ± 1°C. The flow channels are in the center of the basin and are connected with silicone tubes (ø = 1.4 mm) to syringes on both sides. On one side, the blood is pumped back and forth through the channels via a syringe located in a vertically oriented syringe pump. An air cushion of 1 mL prevents contact between the blood sample and the rubber of the syringe plunger, which would cause additional hemolysis during the experiment. On the other side of the channels, open syringes are used as reservoirs. They are covered with 3D‐printed air‐permeable caps to protect the blood from contamination. The unloaded blood volume in the tube per cycle is 0.63 to 0.8 mL. The blood is mixed as it flows into the reservoir. With the setup, up to four flow channels can be examined simultaneously. To detect local hematocrit variations and to qualitatively capture the flow field, high‐speed images of the flow at 5% hematocrit are acquired. For this purpose, the high‐speed camera [MotionXtraN4‐S3, IDT, Newark, New Jersey, USA] is combined with a digital microscope [VHX‐970F, Keyence, Milton Keynes, UK] according to Lommel et al. [16]. To determine the time required for a complete flow reversal during a direction change, which is delayed due to the compliance of the test rig components, the volume flow was evaluated optically by recording the level of the reservoir during several cycles.

### 2.2 Flow Channels

The flow channels were fabricated in quartz glass using the selective laser‐induced etching (SLE) process [17]. The process enables optically accessible 3D structures with an accuracy of 1 to 2 μm and a surface roughness of 1 μm.

**FIGURE 2:**
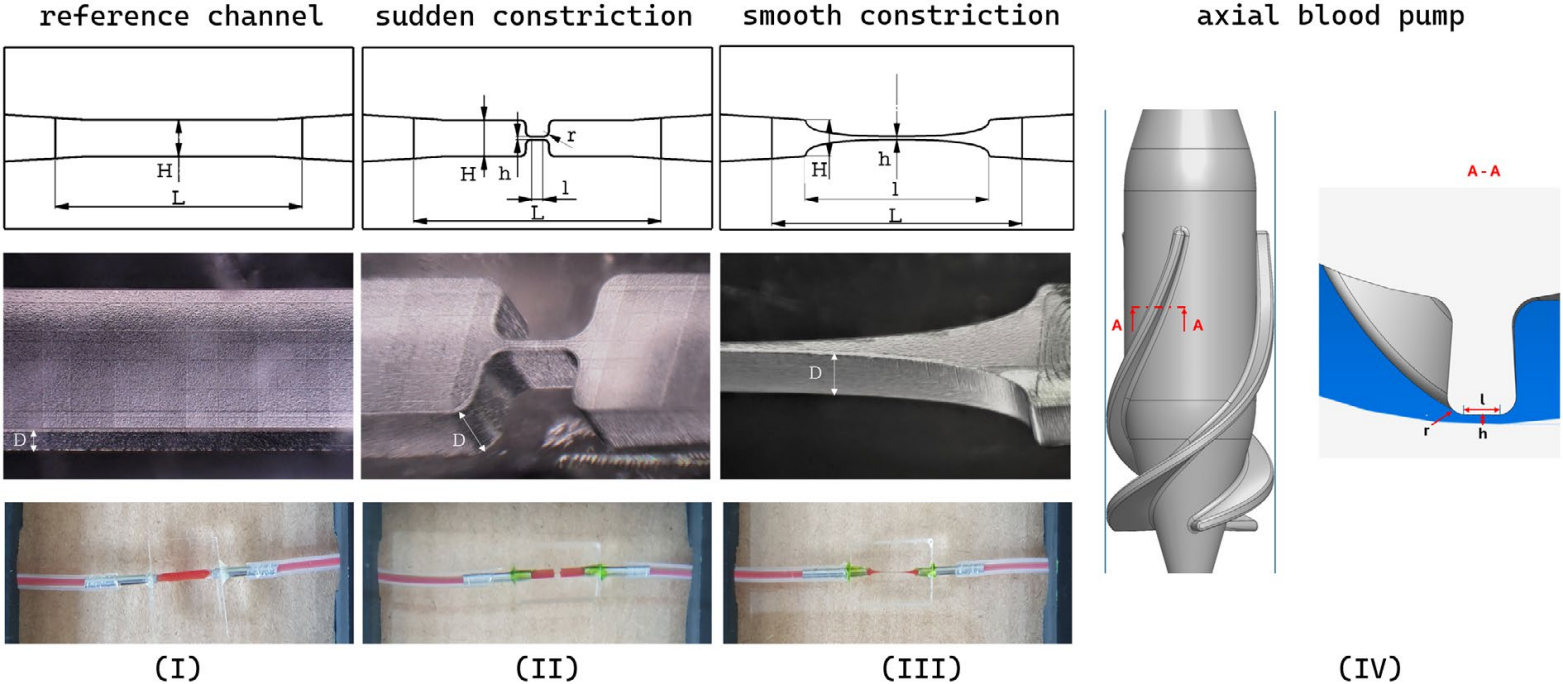
Laser‐etched flow channels used in the experiments. They have a depth (D) of 1 mm, a height (H) of 1.17 mm, and a length (L) of 8.5 mm. Flow channel I is used as a reference channel to measure the hemolysis induced by the test setup. Flow channel II has a constriction height (h) of 101.5 μm over the length (l) of 400 μm. The radius (*r*) at the constriction is 0.2 mm. Flow channel III has a smooth hyperbolic constriction over the length (l) of 6.25 mm with a height (h) of 102 μm at the narrowest point. Below, the optically accessible channels filled with blood are shown during an experiment. IV shows the gap region of the HeartMate II RBP, on which the geometry of channel II was based. [Color figure can be viewed at wileyonlinelibrary.com]

Three different channel geometries were used in the study (see Figure 2). Each had a rectangular cross section with a depth (D) of 1 mm over a length (L) of 8.5 mm. At the inlet and outlet, cannulas with a diameter of 1.3 mm connected the channels with silicone tubes. The inlet and outlet of the channels are in line with the constriction to reduce the hemolytic influences of the RBC‐wall contact. The critical dimensions of the manufactured channels were measured optically from both sides (top and bottom) with a digital microscope with a resolution of 0.3 μm/pixel. The geometries after fabrication were reconstructed for the computational simulations based on the microscopic images. The first channel (I) is the reference channel with no constriction and therefore no increased stresses to measure the hemolysis resulting from the test setup itself. The measured height (H) is 1.180 ± 0.001 mm with a depth (D) of 1 mm.

The second channel (Figure 2, II) has a height (H) of 1.182 ± 0.001 mm and a depth (D) of 1 mm with a symmetric, sudden constriction in the middle. The geometry of the constriction was derived from the blade gap region of the RBP HeartMate II (Abbott Laboratories, Chicago, USA), shown in Figure 2 (IV), as published by Thamsen et al. [14]. This region was chosen because the HeartMate II is one of the most frequently implanted RBPs [14] and it is included (AxVAD2) in the analysis of typical stresses of RBPs by Fraser et al. [15], from which our target stress range is derived.

In this pump, the gap width (h) is 100 μm, the gap length (l) is 400 μm and the rounding has a radius (*r*) of 0.2 mm. The measured constriction height (h) of the manufactured channel is 101.5 μm.

The third channel (Figure 2, III) has a smooth symmetric hyperbolic constriction with a depth (D) of 1 mm over a length (l) of 6.25 mm with a height (H) at the inlet and outlet of 1.195 ± 0.002 mm and at the narrowest point (h) of 101 ± 1 μm. The geometry was chosen so that the central constriction corresponds to channel II, but the inlet and outlet regions result in a smoother and smaller change in flow cross section. This was designed to achieve shear stresses in typical range of RBPs but lower normal stress components due to lower velocity gradients in the direction of flow, in order to investigate the influence of normal stress components on hemolysis, which Faghih et al. [18] considered critical and underestimated.

### 2.3 Short‐Term Stress Peaks in RBPs

The setup is designed to investigate typical stress loads occurring in RBPs. For this purpose, the range of the typical stress loads occurring in RBPs was derived from the publication by Fraser et al. [15], in which the flow fields of five different RBPs were analyzed. The maximum stresses are in the range of 600 to 3060 Pa and the exposure time to stresses above 150 Pa is between 0.3 and 1.4 ms. The maximum stresses occur in the gap between rotor blades and the housing wall ([15], Figure 5). These stress and exposure time ranges were chosen as the target criterion for our flow setup in order to refer to similar stress loads as in RBPs.

### 2.4 Experimental Procedure

At the beginning of the experiment, 7 to 8 mL of the prepared blood is pipetted into each reservoir. In the second step, the blood is withdrawn into the syringe until the entire tube system is filled. After this, it is pumped back and forth a few times to optically check that there are no air bubbles in the channels. In the third step, the first samples (400 μL) are pipetted off and used as baseline for each reservoir to capture the hemolysis caused by the blood collection and filling process. Afterwards, the experiment is started with a volume flow of 24 mL min^−1^ to reach a peak velocity of 6.2 ms^−1^, similar to the HeartMate II [20]. During the experiment, 400 μL blood samples are taken after a fixed number of repetitions.

### 2.5 Blood Sampling

Blood samples were obtained by venipuncture from five healthy male and female volunteers at the age of 24 to 32 with a complete blood count in the physiological range. CPDA‐1 was used as the anticoagulant solution. The samples were stored at 7°C and used within 24 h. To standardize the samples, they were hemodiluted prior to a hematocrit of 30% ± 2% using phosphate‐buffered saline (PBS), according to the standard of the *American Society of Testing and Materials* (ASTM) for blood experiments [21, 22].

### 2.6 Hemolysis Measurement

The plasma is separated by double centrifugation (1015 *g*, 2 × 10 min). 100 μL plasma is pipetted into cuvettes with 1000 μL sodium carbonate solution (0.1% m/V Na_2_CO_3_). To ensure uniform mixing, each sample is vortexed and the absorbance is analyzed photometrically with the method described by Harboe [23]. Additionally, to validate our own hemolysis measurement procedure, three sets of three blood samples each were given to the hospital laboratory *Labor Berlin* (Labor Berlin—Charité Vivantes GmbH, Berlin, Germany) to determine the pfHb. These samples were stressed by pipetting in a preceding study (see Supporting Information). Hemolysis values are given as hemolysis index (IH), the increase in pfHb in relation to total hemoglobin (Hb): $\mathrm{IH}\left[\%\right]=\frac{\Delta \text{pfHb}}{\mathrm{Hb}}\times 100$.

### 2.7 CFD Simulations

Computational simulations were performed using the commercial CFD software STAR‐CCM+ (Siemens Digital Industries Software, Plano, USA). The channels were discretized using a finite volume approach with a polyhedral mesher in the main flow section. An estimation of the error due to the discretization was performed using the method outlined by Roache [24] (see Supporting Information for mesh and mesh and error estimation). An acceptable error was found for a base size of 24 μm and prismatic elements forming six layers on the walls were used to enable a high resolution of the flow gradients. An additional refinement to a cell size of 6 μm and 24 prismatic layers was carried out in the constrictions to accurately capture the high velocity gradients. To ensure a fully developed flow at the inlet, an inflow area with prismatic mesh elements was connected by a conformal interface to the main mesh. An area of the same size was placed behind the channels to ensure that the boundary conditions at the outlet had no influence on the results. The resulting meshes consist of around 6.6 million cells for the sudden constriction channel, 6.5 million cells for the smooth constriction channel, and 3.2 million for the reference channel.

Blood was modeled as a Newtonian fluid with a density of 1050 kg m^−3^ and a dynamic viscosity of 3.1 mPa s for a hematocrit of 30% at a temperature of 37°C [6, 20]. A no‐slip condition was specified for the wall boundaries, while a mass‐flow condition of $\dot{m}=0.42\;g\;s^{-1}$ was set at the inlet in accordance with the experimental setup. A constant pressure was applied at the outlet. A laminar flow regime is assumed for the simulations because the highest Reynolds number in the channels is Re = 246. Before calculating hemolysis, convergence of velocity and pressure was achieved at the start of the final time step, as well as asymptotic convergence of the velocity and its gradient in an unsteady simulation (see Supporting Information for details).

### 2.8 Hemolysis Modeling

The power‐law model (see Equation (1)) with parameters by [25] for human blood ($C=3.458\cdot 10^{-6},\alpha =0.2777,\beta =2.0639$) was used to estimate the amount of hemolysis induced by the channels, as it showed a high agreement with the experimental results in our Couette shearing device [6]:

(2) $$\mathrm{DI}=3.458\cdot 10^{-6}t^{0.2777}\tau^{2.0639}.$$

Since the literature uses different parameter sets depending on the group and application [10], other commonly used parameter sets are investigated using the Lagrangian method.

Since the experiments are not performed with a simple shear flow, we have calculated the representative resultant scalar shear stress $\tau_{r}$ from the tensor components in analogy with the von Mises stress for solids, according to Bludszuweit et al. [19] and corrected by Faghih et al. [26]:

(3) $$\begin{array}{l} \tau_{r}=\left\{C_{n}^{2}\left[\sigma_{\mathrm{xx}}^{2}+\sigma_{\mathrm{yy}}^{2}+\sigma_{\mathrm{zz}}^{2}\right.\right.\\ \left.\;-\left(\sigma_{\mathrm{xx}}\sigma_{\mathrm{yy}}+\sigma_{\mathrm{xx}}\sigma_{\mathrm{zz}}+\sigma_{\mathrm{yy}}\sigma_{\mathrm{zz}}\right)\right]\\ {\left.\;+\;\left(\sigma_{\mathrm{xy}}^{2}+\sigma_{\mathrm{xz}}^{2}+\sigma_{\mathrm{yz}}^{2}\right)\right\}}^{1/2}, \end{array}$$

where $\sigma_{\mathrm{xx}}$, $\sigma_{\mathrm{yy}}$ and $\sigma_{\mathrm{zz}}$ represent normal stresses and $\sigma_{\mathrm{xy}}$, $\sigma_{\mathrm{xz}}$ and $\sigma_{\mathrm{yz}}$ shear stresses. In the classical formulation of Bludszuweit [19], $\sqrt{3}C_{n}=1$, which effectively weighs normal stresses only a third compared to shear stresses. To accommodate the greater deformation of RBCs under normal stress as compared to shear stress, Faghih et al. [26] suggested adjusting the empirical factor to $\sqrt{3}C_{n}=33.79$ [18]. We evaluated both definitions.

A Lagrangian (*D*_*L*_) and an Eulerian (*D*_*E*_) formulation of Equation (2) is used to calculate hemolysis in the simulations. For the Eulerian approach, the transport equation for the linearized damage fraction *D*_*L*_ according to Farinas et al. [27] was used:

(4) $$\left(\frac{\partial }{\partial t}+\underline{v}\cdot \nabla \right)D_{L}=S,$$

where the left side is the total derivative of *D*_*L*_ and the right side is a source term defined as

(5) $$S={\left(C\tau^{\beta }\right)}^{\frac{1}{\alpha }}.$$

After the convergence of the flow simulation, Equation (4) is solved in the entire flow domain. *D*_*E*_ is then calculated by taking the surface average of *D*_*L*_ at the outlet of the domain. *D*_*E*_ is monitored during the simulation and the final value is extracted after the surface average changes < 1% over ten time steps.

It should be noted that the derivation of this Eulerian model is technically valid only for uniaxial flows with constant velocity along streamlines [2]. Since this is not strictly the case in our setup, some modeling error is expected. Since the model (4) has been used by many research groups in the past despite this error, we use it here as well for better comparability [1, 2, 14].

For the Lagrangian approach, the values for shear stress, extensional stress, and exposure time were determined along 10 000 pathlines of massless particles in an unsteady simulation. The particles were seeded on a circular surface near the inlet of the channel geometry with a cell‐free layer of at least 5 μm from the wall. The distribution of the particles on the surface was based on a weighted random distribution, the weighting of which corresponds to the velocity distribution of a Hagen–Poiseuille flow. As a result, more particles are seeded in the center of the channel, which reflects the higher volume flow there. After at least 99% of particles had traversed the domain, the calculation of hemolysis was carried out by differentiating Equation (1) in time alone [28, 29] and then summing the incremental damages along the pathlines:

(6) $$D_{L}=\sum_{i}\Delta D_{i}=\sum_{i}\mathrm{A\alpha}t_{i}^{\alpha -1}\tau_{i}^{\beta }\Delta t_{i},$$

where *t*_*i*_ is the elapsed exposure time between the start of the stress event and the current iteration *i*, Δ*t*_*i*_ is the time step length at iteration *i*, and $\tau_{i}$ is the average shear stress during this time step. This scalar shear stress was calculated from the local velocity gradient using the two different formulations presented above. The average damage from all completed pathlines is then computed to obtain a single representative hemolysis value.

We compare the predictions of this stress‐based hemolysis model to a strain‐based model. For this purpose, we employ the tank‐treading cell deformation model (TTM) [9]. Thereby, RBCs are modeled as ellipsoidal droplets with three semi‐axes $\lambda_{1}>\lambda_{2}>\lambda_{3}$. The cells deform and rotate according to the local flow forces, that is, the strain tensor $E_{\mathrm{ij}}=\frac{1}{2}\left(\frac{\partial u_{i}}{\partial x_{j}}+\frac{\partial u_{j}}{\partial x_{i}}\right)$ and vorticity tensor $W_{\mathrm{ij}}=\frac{1}{2}\left(\frac{\partial u_{i}}{\partial x_{j}}-\frac{\partial u_{j}}{\partial x_{i}}\right)$. The model can be evaluated in an Eulerian and Lagrangian way. For simplicity, we restrict ourselves to the Lagrangian formulation in this work. The Lagrangian formulation represents a system of ordinary differential equations:

(7a) $$\begin{array}{l} \frac{d\lambda_{i}}{dt}=-f_{1}\left(\lambda_{i}-\frac{3\lambda_{1}\lambda_{2}\lambda_{3}}{\lambda_{1}\lambda_{2}+\lambda_{1}\lambda_{3}+\lambda_{2}\lambda_{3}}\right)\\ +2f_{2}\lambda_{i}{\tilde{E}}_{\mathrm{ii}},\;\tilde{E}=Q^{T}\mathrm{EQ} \end{array}$$

(7b) $$Q=\left\{\begin{array}{ll} Q_{⋆}\left(\Lambda ,E,W\right), & \text{tank}‐\text{treading},\\ 0, & \text{tumbling}, \end{array}\right.$$

with coefficients $f_{1}=5.0\;s^{-1}$ and $f_{2}=4.2298\cdot 10^{-4}$. Here, Equations (7a) and (7b) describe the deformation and orientation of RBCs, respectively. The orientation $Q_{⋆}$ is computed such that the rotating moments of strain and vorticity are in equilibrium. The exact procedure is described in Dirkes et al. [9]. The model is evaluated by integrating the equations along the same pathlines as the stress‐based models above. Based on the deformation δ of the RBCs, we compute an effective stress $\tau_{\mathrm{eff}}$:

(8) $$\delta =\frac{\sqrt{\lambda_{1}}-\sqrt{\lambda_{3}}}{\sqrt{\lambda_{1}}+\sqrt{\lambda_{3}}},\;\tau_{\mathrm{eff}}=\frac{2\mu \delta f_{1}}{\left(1-\delta^{2}\right)f_{2}},$$

which is subsequently used in the power‐law model (2) to estimate hemolysis. The Lagrangian hemolysis analysis using stress‐based and strain‐based models is available as part of the open‐source software HemTracer.^1^

Taken that the hemolysis caused by repetitive stress loads is linear in time, if the RBCs have a relaxation time between the loads [6, 30, 31], the DI increase of repeated stress loads is summed up.

## 3 Results

### 3.1 Experiments

**FIGURE 3:**
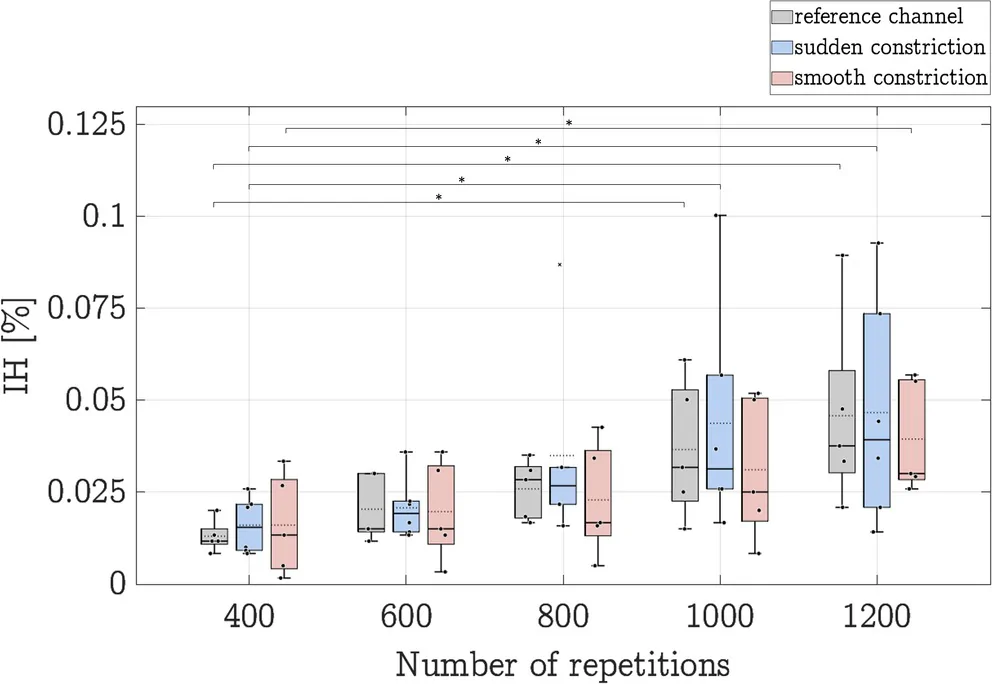
Comparison of the measured hemolysis. The plasma free hemoglobin [mg/dL] was measured and the index of Hemolysis (IH [%]) calculated. There is a significant difference between the 400 and 1200 repetitions with *p* = 0.006 for the reference channel, *p* = 0.024 for the sudden constriction channel, and *p* = 0.028 for the smooth constriction channel, as well as between 400 and 1000 repetitions for the reference channel (*p* = 0.016) and the sudden constriction channel (*p* = 0.014) (Kruskal–Wallis Test). The hemolysis in both channels with constriction does not differ significantly from the reference channel at any number of repetitions. [Color figure can be viewed at wileyonlinelibrary.com]

We collected 80 hemolysis data points. Each flow channel was tested at least five times and at least six samples were taken at 0, 400, 600, 800, 1000 and 1200 repetitions. The hemolysis caused by collection, transport and filling process was subtracted from all values. For this purpose, the sample taken at the beginning of each experiment, on average an IH of 0.01 (±0.07) %, was subtracted from the measuring values. The increase of hemolysis over the number of repetitions is shown in Figure 3. For all three channels, the IH increased significantly from 400 to 1200 repetitions (*Kruskal–Wallis‐Test, SPSS, IBM, USA*). For the reference channel from 0.013% at 400 repetitions to 0.046% at 1200 repetitions (*p* = 0.006), for the sudden constriction from 0.016% to 0.047% (*p* = 0.024) and for the smooth constriction channel from 0.016% to 0.039% (*p* = 0.028) in average.

The hemolysis in both channels with constriction does not differ significantly from the reference channel at any number of repetitions. The average hemolysis caused within all channels does not exceed the pathological threshold value of diagnostic laboratories of 0.05% [32].

The measurement procedure and determination of hemolysis was validated by a simultaneous evaluation with the hospital laboratory *Labor Berlin* (see Supporting Information). Therein, the maximum deviation of the IH is 0.018% and the average deviation is 0.009%.

No separation of cells and plasma can be seen in the high‐speed images, but an attachment of the jet to the wall in the sudden constriction channel behind the constriction was observed (see Figure 5c). The changeover time until the fully developed flow is 2.51 ± 0.098 s, 12.6% of each cycle.

### 3.2 Computational Analysis

**FIGURE 4:**
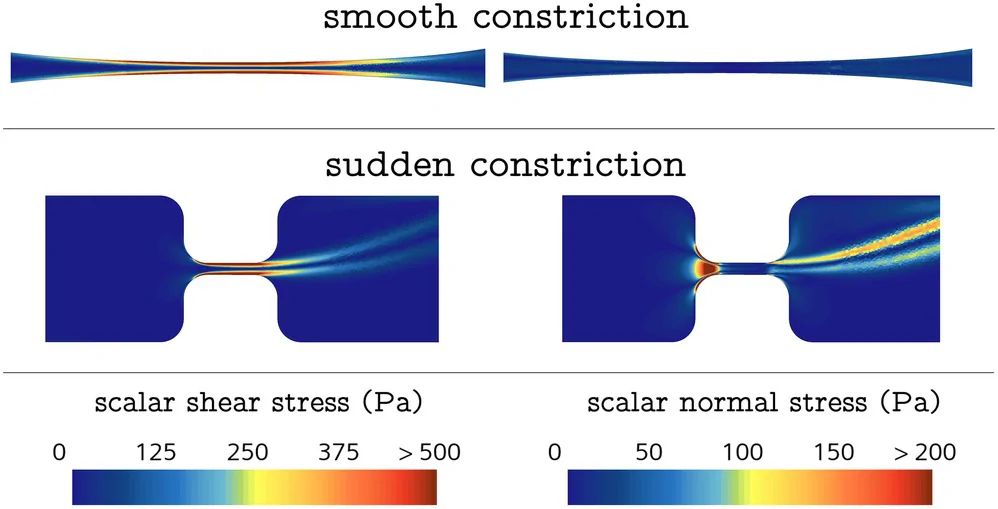
Simulation results of the occurring shear stress and normal stress components of the scalar shear stress $\tau_{r}$ shown in the channels with constriction. The stress scales were cut off at 500 and 200 Pa, respectively, although higher stresses were recorded to better highlight areas of high stress away from the wall. [Color figure can be viewed at wileyonlinelibrary.com]

First, it was tested whether the design of the channels is suitable for differentiating the influence of shear and normal stress components on hemolysis. In Figure 4, the normal and shear stress components of the stress tensor were compared for both channels with constriction (in detail in Supporting Information). The shear stresses in the smooth constriction are up to 800 Pa and in the sudden constriction up to 1350 Pa. The maximum shear stresses in the constrictions are in the derived target range of 600 to 3060 Pa and are therefore comparable to RBPs. The normal stresses in the sudden constriction are up to 871 Pa. In the smooth constriction, they are eight times lower at up to 106 Pa.

**FIGURE 5:**
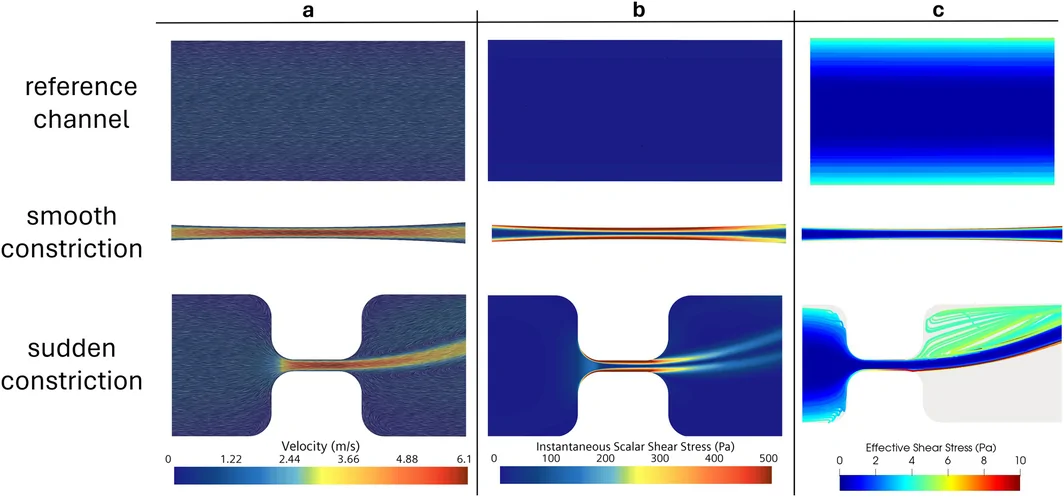
Cross‐sectional view of the simulation results for all three channels. (a) Velocity magnitude in the flow fields. (b) Scalar Shear Stress evaluated according to Bludszuweit [19], cut off at 500 Pa, to highlight areas of high stress in the main flow. (c) Strain‐based model results for the effective shear stress along particle pathlines. [Color figure can be viewed at wileyonlinelibrary.com]

The calculated velocity field, as well as the scalar shear stress field are shown for all three channels in Figure 5. The volume flow of 24 mL min^−1^ leads to a maximum velocity of up to 6.1 ms^−1^ in both channels with constriction (Figure 5a). The flow in the channel with the sudden constriction forms an asymmetric jet behind the constriction as observed in the high‐speed images (Figure 5c). This is consistent with literature, where a flow through a similar geometry creates two stable asymmetric jet configurations under certain flow conditions [33] (see Supporting Information).

**FIGURE 6:**
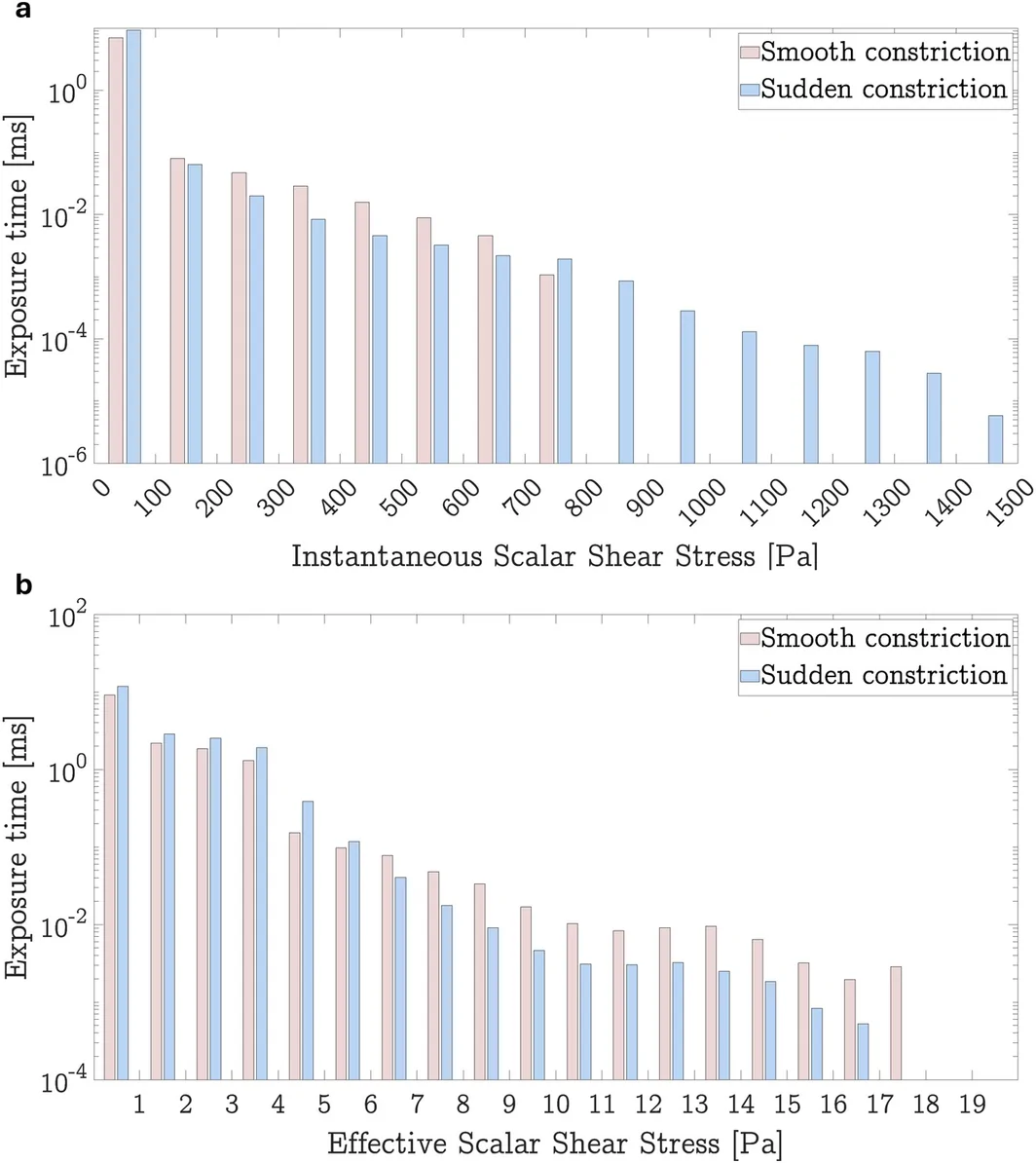
(a) The average exposure time to scalar shear stress levels (definition by Bludszuweit [19]) for one passage through the channels is shown in milliseconds, divided into 100 Pa stress ranges. (b) The effective scalar shear stress levels acting on the membrane calculated from the strain‐based hemolysis model. For the calculation, the time‐dependent deformation of the membrane is taken into account. [Color figure can be viewed at wileyonlinelibrary.com]

In the flow field, the instantaneous scalar shear stress is up to 1500 Pa for the sudden constriction channel and 800 Pa for the smooth constriction channel. Figure 6a shows the average exposure time to different instantaneous scalar shear stress levels in the flow field for the average passage through the channels. The average exposure time to elevated stresses above 15 Pa is 1.2 ms in the smooth constriction channel and 0.9 ms in the sudden constriction channel. The stress levels and the exposure times in the channels with constrictions are therefore within the range determined to be typical of RBPs. In the reference channel, the scalar shear stresses are < 15 Pa.

Figure 6b shows the resulting time‐dependent effective stress acting on the cell membrane calculated in the strain‐based model as a measure of the cell's instantaneous membrane strain. Due to the short exposure times and the considered duration of the membrane deformation, the effective scalar shear stresses are below 18 Pa for all channels and therefore two orders of magnitude lower than the instantaneous scalar shear stresses in the flow field.

The results of the hemolysis models are shown in Table 1 for the average passage and after 1200 passages. The stress‐based models, which are based on instantaneous stresses shown in Figure 6a, predict an IH of several orders of magnitude higher for the channels with constriction than for the reference channel. The empirical factor of Faghih et al. [2] for a stronger weighting of the normal stress components leads to a two orders of magnitude higher hemolysis prediction in the sudden constriction channel using the Lagrangian method and to a 10 orders of magnitude higher and unphysiological hemolysis prediction using the Eulerian method.

**TABLE 1:** Results of the hemolysis model with parameters by Ding et al. [25] in IH [%] for an average passage and 1200 passages compared to the experiments.

	Channel	Experiments	Bludszuweit		Faghih Sharp		TTM
			Lagrange	Euler	Lagrange	Euler	Lagrange
Average passage	Reference	3.6 × 10^−5^	4.1 × 10^−6^	1.4 × 10^−12^	6.5 × 10^−5^	2.2 × 10^−5^	1.6 × 10^−6^
	Smooth	3.2 × 10^−5^	6.7 × 10^−4^	1.4 × 10^−3^	1.4 × 10^−2^	2.1 × 10^−1^	1.5 × 10^−6^
	Sudden	4.1 × 10^−5^	4.7 × 10^−4^	4.2 × 10^−3^	6.4 × 10^−2^	7.5 × 10^6^	1.4 × 10^−6^
Passages	Reference	4.5 × 10^−2^	4.9 × 10^−3^	1.7 × 10^−9^	7.8 × 10^−2^	3.0 × 10^−2^	1.9 × 10^−3^
	Smooth	3.9 × 10^−2^	1.2 × 10^0^	1.7 × 10^0^	1.7 × 10^1^	2.5 × 10^2^	1.8 × 10^−3^
	Sudden	4.7 × 10^−2^	5.6 × 10^−1^	5.0 × 10^0^	7.7 × 10^1^	9.0 × 10^9^	1.7 × 10^−3^

*Note:* The stress‐based damage model with the scalar shear stress definitions of Bludszuweit [26] and Faghih and Sharp [18] and the parameters by Ding et al. [25] were applied using the Lagrangian [29] and the Eulerian [27] method. The strain‐based hemolysis model (TTM) approach presented by [9] was applied.

The strain‐based hemolysis model, which calculates hemolysis based on the effective scalar shear stresses (Figure 6b) predicts with 1.7 × 10^−3^ to 1.9 × 10^−3^ a magnitude lower and similar IH for all three channels after 1200 repetitions.

The investigation of the other commonly used parameter sets of the strain‐based hemolysis model shows similar tendencies and are presented in the Supporting Information.

**FIGURE 7:**
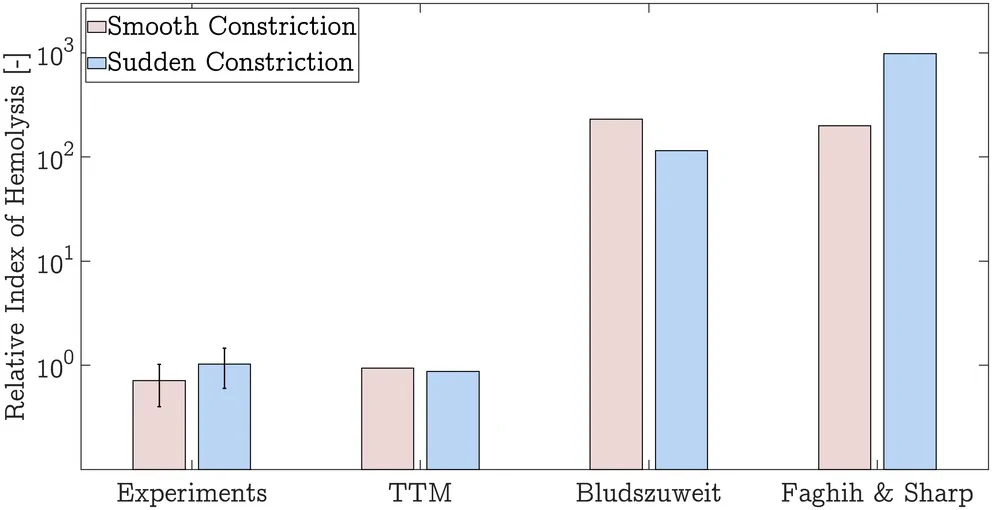
The relative index of hemolysis predicted in the channels with constriction compared to the reference channel was calculated and compared to the experiments to investigate the qualitative predictive power of the models. [Color figure can be viewed at wileyonlinelibrary.com]

To test the qualitative predictive power of the models, the hemolysis occurring in the channels with constriction relative to the reference channel was considered in Figure 7. The stress‐based models predict a several orders of magnitude higher relative IH, while the prediction of the strain‐based model is within the experimental measurement uncertainty and thus corresponds qualitatively.

## 4 Discussion

We developed a novel experimental setup that allows for a high number of stress loads with low hemolysis over a period of 6 h and 1200 load repetitions at 36°C. The setup with the reference channel only increased the IH by 0.048%. For classification, this is a lower hemolysis than after 24 h storage in a blood bag [34] and a significantly higher damage is obtained with 10 times careful pipetting in other studies [35]. It can be operated with small blood volumes of 8 mL per test geometry, and up to four tests can be performed simultaneously with the same drive and blood sample, which leads to a high comparability. Due to the small amount of fluid required, the experiments can be performed with human whole blood. The optical accessibility of all blood‐carrying components enables additional optical controls and evaluation options.

The setup and channels were designed in such a way that the load is comparable to critical areas in RBPs. The dimensions of the constriction are similar to the blade gap of the HeartMate II RBP. Thamsen et al. [14] have shown that this region is identified as the main cause of hemolysis according to strain‐based hemolysis models.

In RBP in vitro test stands, the blood is loaded about every 6 s with 500 mL blood and a volume flow of 5 L min^−1^ [22], in our channels every 15 to 20 s, so three times less stress loads occur. Nevertheless, in our channel, all passing blood experiences high stresses, while in blood pumps only a small percentage enters high stress regions, as shown by Fraser et al. [15]. The applied hemolysis models therefore predict a high hemolysis for our channels with constriction. The two constriction geometries allow the differentiation of the influence of shear and extensional stress on hemolysis, as shown in Figure 4 and in the Supporting Information. The latter is thought to have a higher impact on hemolysis than previously estimated [18]. After 1200 repetitions, a statistically significant increase in hemolysis was measured. It is notable that the hemolysis after 1200 repetitions does not differ from the reference channel.

The commonly used stress‐based models based on instantaneous stresses overestimate hemolysis in the constriction channels depending on the method by a factor of up to 106 and were also qualitatively wrong, as shown in Figure 7. The stronger weighting of the short‐term acting normal stress by the empirical factor of Faghih et al. [2] leads to an even greater overestimation of the hemolysis.

An explanation for the overestimation could be the short exposure times for stresses above 15 Pa of 1.2 ms in the smooth constriction channel and of 0.9 ms for the sudden constriction channel. The stress peaks in the RBPs are outside of the range of conditions for which the hemolysis model coefficients were calibrated. The investigation of the FDA nozzle by Mantegazza et al. [13] has shown that even parameter sets calibrated for the specific flow case lead to highly inaccurate hemolysis predictions, as the stress‐based model is entirely empirical and does not include any physics‐based mechanisms such as the time‐dependent deformation of the viscoelastic RBC. The stress‐based models assume an immediate cell distortion at a certain scalar shear stress level while the real cell deformation and relaxation take a certain amount of time. Guglietta et al. [36] present an overview of computational and experimental investigation of time‐dependent deformation. This is supported by the review by Faghih and Sharp [2], who showed an overview of the logarithmic increase in the hemolytic threshold with shorter exposure times for multiple studies. The results are consistent with those of Zhao et al. [12], who applied one‐time shear loads of up to 5170 Pa in a similar microchannel setup and observed no ruptures of RBCs and the results of Down et al. [37] which propose an extensional stress threshold value of 3000 Pa for hemolysis if the exposure times are on the order of microseconds. This would explain the poor hemolysis prediction in our test case and in RBPs, as shown by Ponnaluri et al. [4], because short‐term high shear peaks occur on a large scale in these flows, as shown by Fraser et al. [15].

Faghih and Sharp [38] have pointed out the weaknesses of the Eulerian stress‐based model. It lacks dependence on the duration of exposure to stresses, and the mathematical formulation restricts the applicability to complex flows. The Eulerian form of the strain‐based model, as described by Dirkes et al. [9] eliminates some of these shortcomings.

The strain‐based hemolysis model presented is physics‐based, as it resolves the shape distortion of RBCs over time according to their relaxation time [9]. The occurrence of hemolysis is attributed to the membrane strain of the RBCs. Thereby, the model accounts for the cell membrane's viscoelastic behavior. The resulting effective stress is a measure of the cells' instantaneous membrane strain. In the short‐term loads at hand, the membrane does not have enough time to deform significantly in response to the flow forces. As a result, the effective stress levels are much lower than the instantaneous stresses used in the stress‐based models (Figure 6). The results of the model agree qualitatively with the experiments, as it predicts almost equal and low IH in all three channels. Quantitatively, the IH values are an order of magnitude below the experiments, in the range below the hemolysis measurement accuracy. Since the total hemolysis of the entire test stand is measured experimentally with additional effects such as aging of the RBCs and blood handling, predicted hemolysis below or in the range of the hemolysis caused by the reference setup cannot be determined. So, the predicted values are plausible, since the hemolysis in the channels does not differ significantly from the reference channel. With the experiments, we were able to demonstrate the limited applicability of the commonly used stress‐based hemolysis models for our flow case. The presented strain‐based hemolysis model seems preferable for these types of stress loads.

Nevertheless, hemolysis does occur in the clinical use of RBPs [39]. Lommel et al. [6] have shown the validity of the stress‐based hemolysis model with the parameter set by Ding et al. [25] for longer stress exposure times of around 24 ms. Consequently, it is hypothesized that lower stress levels with longer exposure times in the range of 0.01 to 0.1 s in other RBP regions are responsible for the experimentally measured hemolysis. This has also been suggested by Pauli et al. [40], who identified differences in the critical regions predicted by stress‐based and strain‐based models and emphasized the importance of the combination of instantaneous shear and exposure time. This hypothesis leads to different optimization criteria for blood pumps than are currently the case.

To further investigate this, we are manufacturing a 200 mm long flow channel and designing a novel pressure‐driven setup to be able to investigate similar stresses but with exposure times of up to 0.3 s.

### 4.1 Limitations

This study was performed with a hematocrit of 30% in accordance with the previous standard of the ASTM [22]. The new standard practice [41], which recommends adjusting the hematocrit to 35%, was published after the start of the study. The hematocrit must be adjusted by dilution with PBS or plasma. This study adjusted the hematocrit with PBS, which could influence the blood rheology by reducing the viscoelastic behavior of blood [42]. Moreover, the choice of anticoagulant may affect the outcome of shear‐induced hemolysis but was also not considered in this study. CPDA‐1 was used as an anticoagulant. Paul et al. [43] measured less hemolysis in citrated blood than in heparinized blood under the same shear conditions.

## 5 Conclusion

We measured hemolysis in channels whose geometric constriction induces short‐term high stress peaks in the millisecond range with 1200 repetitions. Only a small amount of hemolysis occurred, with no significant difference between the constricted channels and the reference channel. CFD simulations were performed to test the predictive power of different hemolysis models. The stress‐based hemolysis models used with the Eulerian and Lagrangian approaches estimated significantly higher hemolysis and were qualitatively incorrect in predicting differences between the geometries. The strain‐based hemolysis model prediction was qualitatively correct and quantitatively plausible. The study suggests that stress‐based models do not capture all relevant parameters and therefore have limited applicability to complex flows with stress peaks as they occur in RBPs. Therefore, the presented strain‐based hemolysis model seems to be preferable for these types of stress loads.

## Author Contributions

Conceptualization: Michael Lommel and Vera Charlotte Lommel. Data curation: Michael Lommel, Vera Charlotte Lommel, Henri Wolff, and Nico Dirkes. Formal analysis: Michael Lommel, Henri Wolff, and Nico Dirkes. Investigation: Michael Lommel and Vera Charlotte Lommel. Methodology: Michael Lommel, Vera Charlotte Lommel, Nico Dirkes, and Katharina Vellguth. Supervision: Michael Lommel and Katharina Vellguth. Validation: Michael Lommel. Visualization: Michael Lommel and Vera Charlotte Lommel. Writing – original draft: Michael Lommel, Vera Charlotte Lommel, Henri Wolff, Nico Dirkes. Writing – review and editing: Michael Lommel, Marek Behr, Ulrich Kertzscher, and Katharina Vellguth. Project administration: Michael Lommel, Marek Behr, and Ulrich Kertzscher; Funding acquisition: Marek Behr and Ulrich Kertzscher; Resources: Marek Behr and Ulrich Kertzscher.

## Ethics Statement

The study was approved by the ethics committee of the Charité—Universitätsmedizin Berlin (EA2/279/20, 9.12.2020).

## Conflicts of Interest

The authors declare no conflicts of interest.

## Supporting information


Data S1.

Data S2.

## Data Availability

The data that support the findings of this study are available from the corresponding author upon reasonable request.

## Endnotes

^1^

https://github.com/nicodirkes/HemTracer.
