# Supplementary material for: Experimental Investigation of the Applicability of the Stress‐Based and Strain‐Based Hemolysis Models for Short‐Term Stress Peaks Typical for Rotary Blood Pumps
Source: Artif Organs. 2025 Apr 16;49(7):1108–18. doi: 10.1111/aor.15002 (PMC12179753; doi:10.1111/aor.15002)
Supplement: Supplementary file 1 — Data S1. [file AOR-49-1108-s002.pdf]

Supplement 1: HeartMate II gap geometry

The channel geometry with constriction is based on the blade gap geometry of the RBP Heartmate II (Abbott Laboratories, Chicago, U.S.A). High, short-term stress peaks occur in this region, which are considered by current hemolysis models to be primarily responsible for the hemolysis that occurs in the pump [1]. With a gap length of $l = 0.4\,\mathrm{mm}$, a gap width of $h = 100\,\mu\mathrm{m}$ and a rounding at the blade edge of $r = 0.2\,\mathrm{mm}$, the dimensions correspond to the flow channel with the sudden constriction.

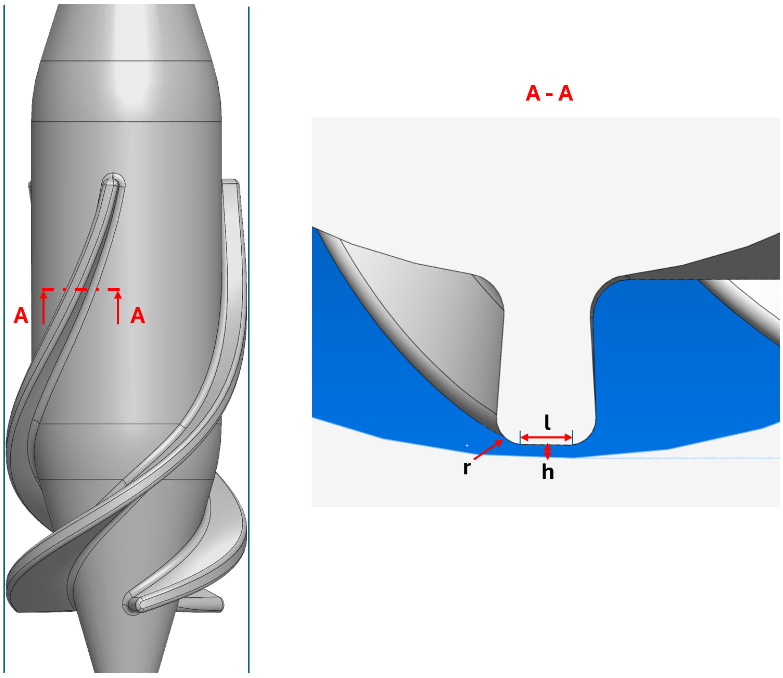

Figure 1: Gap region of the HeartMate II rotor with a gap length of $l = 0.4\,\mathrm{mm}$, a gap width of $h = 100\,\mu\mathrm{m}$ and a rounding at the blade edge of $r = 0.2\,\mathrm{mm}$, the dimensions correspond to the channel with the sudden constriction.

Supplement 2: Hemolysis Measurement Validation

To validate our own hemolysis measurement procedure and measure the hemolysis caused by multiple pipetting, three sets with three blood samples each were given to the hospital laboratory *Labor Berlin* (Labor Berlin - Charité Vivantes GmbH, Berlin, Germany) to determine the fHb. Each set consisted of an almost undamaged sample taken by pipetting 500 µL once with a pipette tip for 1000 µL pipetting. And two samples of 500 µL that were pipetted back and forth between two 1.5 mL sample tubes five and ten times, respectively. The fHb value of the first sample was subtracted from the other two samples to eliminate the hemolysis caused by the longer storage time of the sample and transport to the laboratory. The measurement procedure and determination of hemolysis was validated in a preceding study by a simultaneous evaluation with the hospital laboratory *Labor Berlin* see Table 1. Therein, the maximum deviation of the fHb is $1.8\,\mathrm{mg\,dL^{-1}}$ and the average deviation is

0.009 mg dL^{-1}. The average hemolysis (n=3) caused by pipetting 5 times was 5.1 (±3.31) mg dL^{-1} and by pipetting 10 times 10.37 (±2.55) mg dL^{-1}.

		Own measurement		Labor Berlin	
	Number of pipetting steps	fHb [mg/dl]	corrected fHb [mg/dl]	fHb [mg/dl]	corrected fHb [mg/dl]
A	1	0.3	**0.0**	2	**0**
	5	4.7	**4.4**	6	**4**
	10	8.1	**7.8**	8	**6**
B	1	5.5	**0.0**	6	**0**
	5	7.6	**2.2**	9	**3**
	10	15.8	**10.4**	18	**12**
C	1	4.1	**0.0**	5	**0**
	5	12.8	**8.7**	14	**9**
	10	17.0	**12.9**	18	**13**

Table 1: Validation of the hemolysis measurement procedure. The hemolysis measurement of three sets (A-C) each consisting of three samples has been measured with the above-described procedure and additionally submitted to the diagnostic laboratory *Labor Berlin - Charité Vivantes GmbH, Berlin, Germany* for evaluation.

Supplement 3: Devices and Materials

Thermostat	Haake Thermo C1, Thermo Fisher Scientific, Waltham, USA
Syringes	Plastipak 50mL, Becton Dickinson, Franklin Lakes, USA
Syringe pump	Phd Ultra cp,Harvard Apparatus, Cambridge, USA
Blood reservoir	BD Plastipak 10mL, Becton Dickinson, Franklin Lakes, USA
Silicone glue	Elastosil 601, Wacker Chemie AG, Munich, Germany
Digital microscope	VHX-7000, Keyence, Osaka, Japan
Anticoagulant	S-Monovette CPDA-1 8.5 mL, Sarstedt, Nümbrecht, Germany
PBS	Dulbecco's Phosphate Buffered Saline, Life Technologies, Carlsbad, USA
Cuvettes	Semi-micro cuvette, 3 mL, Sarstedt, Nümbrecht, Germany
Sodium Carbonate	Sodium carbonate anhydrous A1881, AppliChem GmbH, Darmstadt, Germany
Photometer	Ultrospec 3000,Pharmacia GmbH, Erlangen, Germany
Pipette tip	Pipette tip 1000 µl, Sarstedt, Nümrecht, Germany

Table 2: Devices and Materials

Supplement 4: Impinging jet in the sudden constriction channel

The flow in the channel with the sudden constriction forms an asymmetric jet behind the constriction in the simulations as it is also observed in the high speed images of the experiments which were used to verify the uniform hematocrit (Figure 2).

This phenomenon is consistent with the literature, where a flow through a constriction creates two stable flow configurations under certain flow conditions: One with the jet impinging on the upper wall and one with it impinging on the lower wall [2]. The symmetric jet configuration is unstable after this bifurcation. This so-called supercritical pitchfork bifurcation occurs at a critical Reynolds number Re_c that not only depends on a reference length, the viscosity of the liquid and the volume flow, but also on the ratio of the channel height in the constriction to the channel height in the expansion behind it [3]. Fearn et al. [2] calculated a critical Reynolds number near 33 for their setup, which had a sharp 90 degree expansion and an expansion ratio of 1:3. They based the Reynolds number on the maximum inlet velocity and the channel half-height in the constriction. Using these reference parameters, our simulation is carried out at a Reynolds number of 103 for the sudden constriction channel. This suggests that the symmetric jet configuration is unstable in our setup. Cherdron et al. [3] discuss the influence of the expansion ratio in their work and conclude that Re_c decreases as the expansion ratio increases. As we have a much higher expansion ratio of around 1:11.5, we conclude that our chosen Reynolds number is sufficiently high to produce stable asymmetric flow configurations in our CFD simulations and experimental setup.

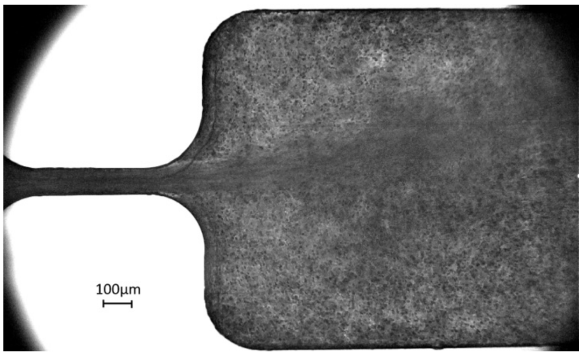

Figure 2: High-speed images of the slit with sudden constriction at 5000 fps. The images were used to verify a uniform hematocrit and to capture the asymmetric Jet.

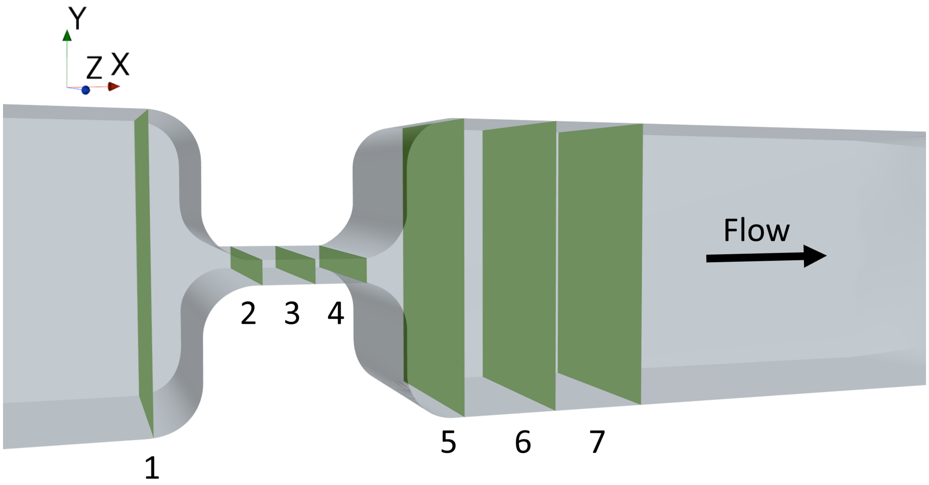

Figure 3: Cross-sectional planes (green) used to assess convergence for the simulations. For each monitored variable, the surface average was calculated each time step and checked for convergence across ten time steps.

Supplement 5: Mesh Independence Study

To investigate mesh convergence, we examined several characteristic parameters of the flow field for different base sizes. The study was conducted exemplary using the sudden constriction geometry, as it has the highest velocity gradients and thus requires the finest mesh of all three geometries.

Convergence of a simulation was assessed in part by verifying that the residuals of the continuity and momentum equations were less than 10^{-4} at the start of the final time step. Additionally, we ensured convergence of the velocity field and its important derivatives along seven cross-sectional planes in the channel geometry (see Figure 3) by monitoring their surface average on these planes at each time step. The pressure drop across the geometry was monitored as well. Simulations were declared as converged only after a relative change of less than one percent was measured for each variable for at least ten time steps, if the residuals had converged as well.

For the mesh independence study, we compared the base sizes 16 µm, 24 µm and 32 µm. In the constriction region, the base size is refined to 25 % to better capture the high gradients. This gives a refinement ratio r of 1.5 between the coarse and the medium mesh, as well as between the medium and the fine mesh. Table 3 includes more information on the three meshes used.

An error estimation was performed using the method outlined by Roache [4]. For that, a probe grid was placed inside the constriction and the convergence of several parameters was assessed there. We chose to assess convergence here, as the highest velocity gradients and thus the highest hemolysis is expected in this region. The grid used can be seen in Figure 4. The parameters used to assess mesh independence were the respective L2 norms of the vectors containing the values of the following parameters for each grid point: the velocity component in the main stream direction

Parameter		Meshes		
Mesh Name		Coarse	Medium	Fine
Base Size in um		32	24	16
Refined Prism Layer Parameters	Near Wall Layer Thickness in µm	1.125	0.75	0.5
	Stretching Ratio	1.095	1.0625	1.04
	Prism Layer Thickness in µm	38.7	39.4	38.8
Default Prism Layer Parameters	Near Wall Layer Thickness µm	4.5	3.0	2.0
	Stretching Ratio	1.5225	1.2925	1.1775
	Prism Layer Thickness in µm	37.7	37.6	37.8
Total Number of Cells		2,074,154	6,627,903	21,462,522

Table 3: Base sizes, prism layer parameters and the corresponding total number of cells for the sudden constriction geometry of all three meshes used during the mesh independence study.

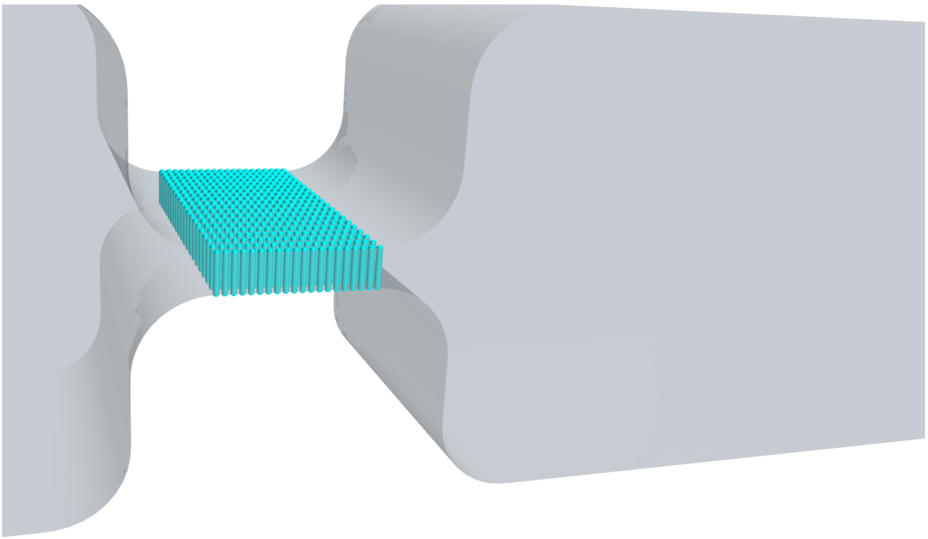

Figure 4: Probe grid used to extract parameters from the simulations during the mesh independence study. The grid covers a volume where high shear rates occur.

(x coordinate, see Figure 3), the velocity component in the y direction and the derivative of the velocity in x direction w.r.t. y, which becomes important near the outlet of the constriction due to the asymmetrical jet configuration. Additionally, we checked convergence of the instantaneous scalar stress (called τ here).

First, the observed order of convergence p was calculated with

$$p = \frac{\log\left(\frac{|\Phi_{\text{coarse}} - \Phi_{\text{medium}}|}{|\Phi_{\text{medium}} - \Phi_{\text{fine}}|}\right)}{\log r}, \tag{1}$$

where Φ is a parameter obtained in either the coarse, medium or fine mesh of the sudden constriction and r is the refinement ratio.

Next, the discretization error E of the parameter can be estimated with

$$E_{\text{coarse}} = \frac{r^p \frac{|\Phi_{\text{coarse}} - \Phi_{\text{medium}}|}{|\Phi_{\text{coarse}}|}}{r^p - 1}, \tag{2}$$

$$E_{\text{medium}} = \frac{\frac{|\Phi_{\text{coarse}} - \Phi_{\text{medium}}|}{|\Phi_{\text{coarse}}|}}{r^p - 1}, \tag{3}$$

$$E_{\text{fine}} = \frac{\frac{|\Phi_{\text{medium}} - \Phi_{\text{fine}}|}{|\Phi_{\text{medium}}|}}{r^p - 1}. \tag{4}$$

The results of this analysis can be found in table 4. While the solver we use has a theoretical order of convergence of two, the observed order of convergence differs from that theoretical maximum.

While the velocity in the main stream direction (u_x) and its derivative ($\partial u_x/\partial y$) show good order of convergence between one and two, the transverse in-plane velocity component (u_y) barely converges and thus has a higher error associated to it. This was deemed as acceptable since the biggest velocity gradients in magnitude are associated with the main stream velocity component.

τ exhibits a sign of super-convergence, as the observed value of convergence is higher than the theoretical value of 2. This means that the estimated error values are not reliable for this variable [5].

The medium mesh offers a good balance between estimated errors and computational time. For this reason, it was chosen for this geometry and the parameters for its contruction were used as a basis for the other two geometries. The resulting mesh for a base size of 24 µm can be seen in Figure 5.

Parameter	Order of Convergence	L2 Norm (Estimated Error in %)		
		Coarse	Medium	Fine
u_x	1.80	$3.65\,\mathrm{m\,s^{-1}}$ (1.76)	$3.71\,\mathrm{m\,s^{-1}}$ (0.85)	$3.68\,\mathrm{m\,s^{-1}}$ (0.41)
u_y	0.18	$0.0248\,\mathrm{m\,s^{-1}}$ (10.16)	$0.0251\,\mathrm{m\,s^{-1}}$ (9.44)	$0.0247\,\mathrm{m\,s^{-1}}$ (8.84)
$\partial u_x/\partial y$	1.34	$1.167 \times 10^5\,\mathrm{s^{-1}}$ (4.41)	$1.150 \times 10^5\,\mathrm{s^{-1}}$ (2.56)	$1.168 \times 10^5\,\mathrm{s^{-1}}$ (1.51)
τ	5.02	750.4 Pa (0.99)	739.0 Pa (0.13)	743.2 Pa (0.02)

Table 4: Results of the mesh convergence study: Observed order of convergence and estimated error for each parameter considered.

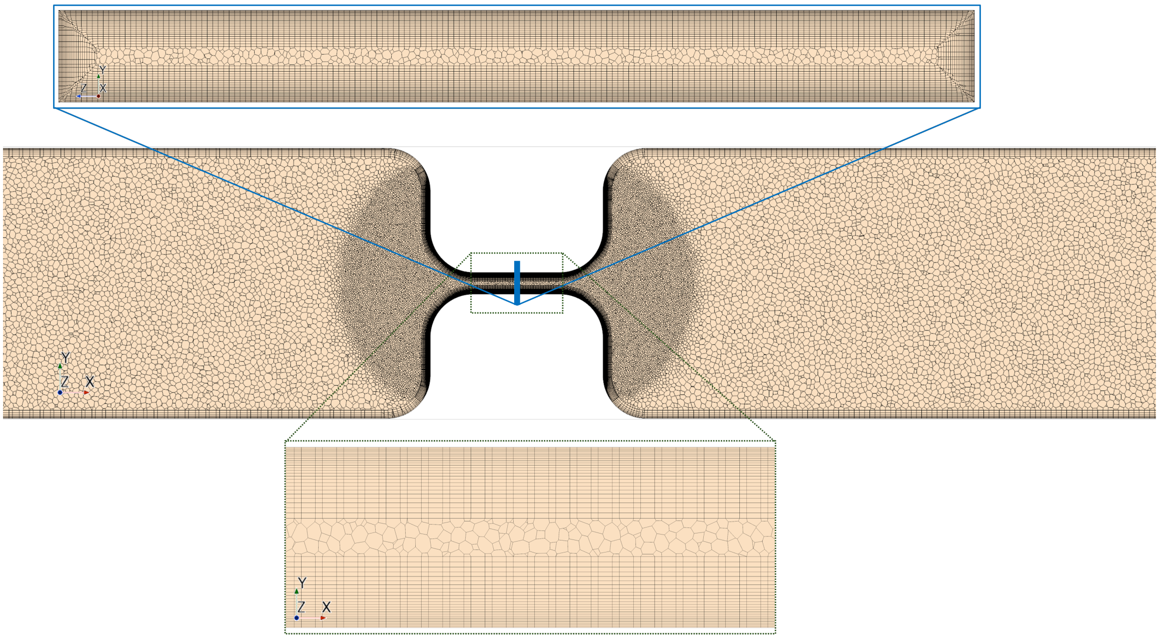

Figure 5: Cross-sectional view of the final mesh (parameters in Table 3 under *Meshes: Medium*). The important features of the polyhedral mesh include the use of prism layers near the walls and a refinement of 25% inside the sudden constriction.

Supplement 6: Alternative Hemolysis Correlations

In table 5 are the results with the parameter sets most commonly used in the literature for the stress-based hemolysis models. The results differ in the absolute amount, but not in the trend. This indicates that the incorrect prediction in the investigated flow case is independent of the chosen parameter set.

Table 5: Comparison between commonly used hemolysis correlations evaluated for the average passage using Lagrangian hemolysis modeling.

Channel	Experiments	Correlation	Bludszuweit	Faghih & Sharp	TTM
Reference	3.6×10^{-5}	Ding	4.1×10^{-6}	6.5×10^{-5}	1.6×10^{-6}
		Zhang	6.5×10^{-6}	7.1×10^{-5}	2.2×10^{-6}
		Giersiepen	2.5×10^{-5}	4.0×10^{-4}	6.9×10^{-6}
		Heuser	7.4×10^{-7}	7.4×10^{-6}	2.4×10^{-7}
Smooth	3.2×10^{-5}	Ding	9.7×10^{-4}	1.4×10^{-2}	1.5×10^{-6}
		Zhang	1.1×10^{-3}	1.4×10^{-2}	1.7×10^{-6}
		Giersiepen	2.8×10^{-2}	3.7×10^{-1}	6.4×10^{-6}
		Heuser	1.2×10^{-4}	1.5×10^{-3}	1.9×10^{-7}
Sudden	4.1×10^{-5}	Ding	4.7×10^{-4}	6.4×10^{-2}	1.4×10^{-6}
		Zhang	5.8×10^{-4}	6.4×10^{-2}	1.7×10^{-6}
		Giersiepen	1.5×10^{-2}	5.1	5.4×10^{-6}
		Heuser	6.5×10^{-5}	7.2×10^{-3}	1.8×10^{-7}

Supplement 7: Normal and shear stress components in the flow channels

In order to examine whether the setup with the two flow channels with constriction is suitable for determining hemolysis caused by two different types of stress, the normal stress and shear stress components of the scalar shear stress (equation 3) were calculated from the path lines of the simulation. We defined a scalar stress representing the shear stress from the definition of the scalar shear stress as

$$\tau_s = \sqrt{\sigma_{xy}^2 + \sigma_{xz}^2 + \sigma_{yz}^2}, \tag{5}$$

as well as a scalar representing the normal stress:

$$\tau_n = \sqrt{\sigma_{xx}^2 + \sigma_{yy}^2 + \sigma_{zz}^2 - (\sigma_{xx}\sigma_{yy} + \sigma_{xx}\sigma_{zz} + \sigma_{yy}\sigma_{zz})}. \tag{6}$$

These definitions were chosen according to the respective parts in (Equation 3) that are weighted differently in Bludszuweit's as well as Faghih and Sharp's definitions.

Figures 6 and 7 clearly show the distinct stress regimes to which red blood cells are subjected in both constriction channels. The shear stresses in the smooth constriction are up to 800 Pa and 1350 Pa in the sudden constriction and therefore in the typical range of blood pumps 600 Pa to 2500 Pa [6]. The normal stresses in the sudden constriction are up to 871 Pa, thus also in the range of RBPs and 106 Pa in the smooth constriction, which is six times lower than the range.

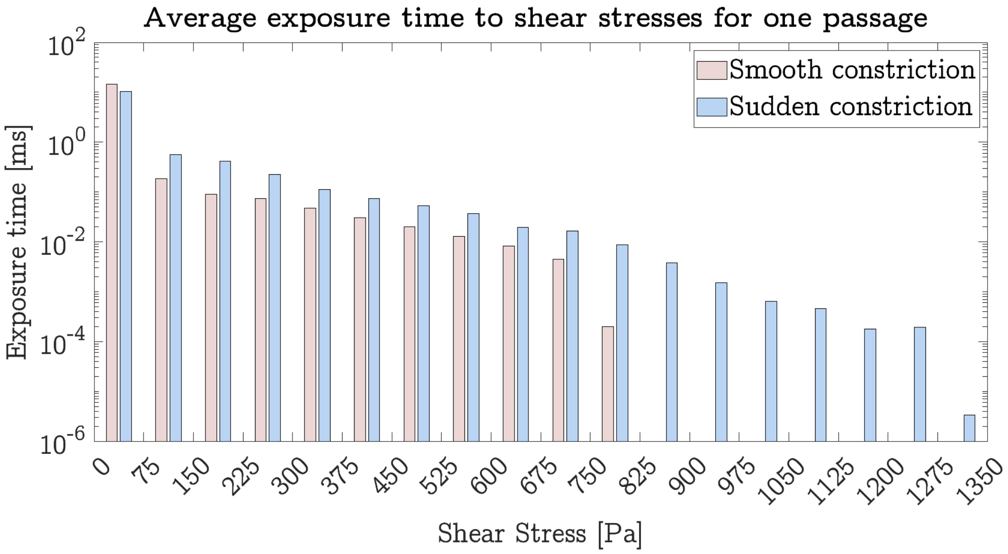

Figure 6: Illustration of the average exposure time to different shear stress levels during a passage through the channels.

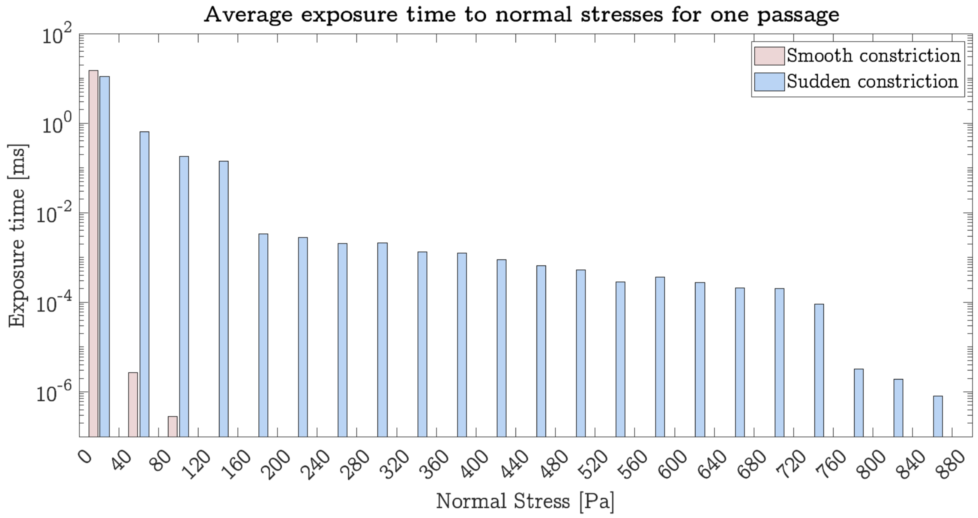

Figure 7: Illustration of the average exposure time to different normal stress levels during a passage through the channels.

References

[1] Bente Thamsen, Bastian Blümel, Jens Schaller, Christian O. Paschereit, Klaus Affeld, Leonid Goubergrits, and Ulrich Kertzscher. Numerical analysis of blood damage potential of the heartmate ii and heartware hvad rotary blood pumps. Artificial Organs, 39:651–659, 8 2015. ISSN 15251594. doi: 10.1111/aor.12542.

[2] R. M. Fearn, T. Mullin, and K. A. Cliffe. Nonlinear flow phenomena in a symmetric sudden expansion. Journal of Fluid Mechanics, 211:595–608, 1990. doi: 10.1017/S0022112090001707.

[3] W. Cherdron, F. Durst, and J. H. Whitelaw. Asymmetric flows and instabilities in symmetric ducts with sudden expansions. Journal of Fluid Mechanics, 84(1):13–31, 1978. doi: 10.1017/S0022112078000026.

[4] P. J. Roache. Quantification of uncertainty in computational fluid dynamics. Annual Review of Fluid Mechanics, 29(Volume 29, 1997):123–160, 1997. ISSN 1545-4479. doi: https://doi.org/10.1146/annurev.fluid.29.1.123. URL https://www.annualreviews.org/content/journals/10.1146/annurev.fluid.29.1.123.

[5] L. Eça and M. Hoekstra. Evaluation of numerical error estimation based on grid refinement studies with the method of the manufactured solutions. Computers & Fluids, 38(8):1580–1591, 2009. ISSN 0045-7930. doi: https://doi.org/10.1016/j.compfluid.2009.01.003. URL https://www.sciencedirect.com/science/article/pii/S0045793009000048.

[6] Katharine H. Fraser, Tao Zhang, M. Ertan Taskin, Bartley P. Griffith, and Zhongjun J. Wu. A quantitative comparison of mechanical blood damage parameters in rotary ventricular assist devices: Shear stress, exposure time and hemolysis index. Journal of Biomechanical Engineering, 134(8), August 2012. doi: 10.1115/1.4007092. URL https://doi.org/10.1115/1.4007092.
